# Long-Term Evolution of Vestibular Compensation, Postural Control, and Perceived Disability in a Population of Patients with Vestibular Neuritis

**DOI:** 10.3390/jcm11143941

**Published:** 2022-07-06

**Authors:** Jonathan Esteban-Sanchez, Eduardo Martin-Sanz

**Affiliations:** 1 Department of Otolaryngology, University Hospital of Getafe, Carretera Toledo km 12,500, 28905 Madrid, Spain; emartinsanz@gmail.com; 2 Department of Medicine, School of Biomedical Sciences and Health, European University of Madrid, C. Tajo, s/n, 28670 Madrid, Spain

**Keywords:** vestibular neuritis, vestibular compensation, disability, unsteadiness

## Abstract

Objectives. The aim was to analyze and compare the compensatory process, vestibular dysfunction, postural control, and perceived disability in a population of patients with vestibular neuritis (VN). Material and Methods. This is a prospective and longitudinal study of 67 patients diagnosed with VN. Inclusion criteria were sudden onset of vertigo, unidirectional spontaneous horizontal nystagmus, and impairment in vestibular test. Exclusion criteria were imaging or clinical findings of any neurotologic disorder. All vestibular tests were performed; vHIT, vestibular evoked myogenic potentials (VEMPs), caloric test and computerized dynamic posturography (CDP), dizziness handicap inventory (DHI), and visual analogue scale (VAS) were also performed at every follow up. Results. We observed a correlation between the composite score of CDP and baseline vestibular function elicited either by caloric test, VEMPs, or vHIT. There was a significant correlation between baseline vestibular function and first visit questionnaire scores. The main gain recovery for the horizontal canal was 0.1 ± 0.04 for the first three months. After that, the gain recovery significantly decreased. The presence of covert and overt saccades’, latency and amplitude decreased, respectively, after the 6-month period, when compared to the baseline results. We also observed a decrease in the PR score from 3 months after the vestibular insult until the last follow up. We observed a significant decrease in DHI and VAS from the first visit until the last one. Those patients with an initial HC gain below 0.5 had significantly higher DHI and VAS scores at every follow up. Conclusions. There are different measurements that could become a complete measurement of the state of compensation, postural control, and disability of the patients. There is a time window in which the vestibular restoration could give us clinical insights regarding the management of VN patients.

## 1. Introduction

Vestibular neuritis (VN) is the main cause of acute vestibular syndrome, which causes long-lasting, continuous, and spontaneous vertigo. It is assumed to be of viral origin [1], because of possible reactivation of latent herpes virus simplex type 1. The main alternative cause postulated is vascular ischemic origin, a theory based on the recurrent finding that vascular risk factors are more prominent in these patients [2]. The viral hypothesis is more contrasted because, currently, there is genetic and radiological evidence of inflammation in the acute phase of VN [3,4].

Simply based on symptoms, VN presentation overlaps with that of a great variety of disorders such as a potentially serious ischemic stroke or minor gastroenteritis [5]. Therefore, the diagnosis should be based on clinical history and physical examination, following the HINTS protocol [6].

The horizontal and superior semicircular canals are more commonly affected in VN [7]. However, in a minority of patients, a complete or a selective involvement of the posterior canal takes place [8]. Although a major vestibular dysfunction in VN is expected, recent studies report some cases with a final diagnosis of possible vestibular neuritis with mild or no objective deficit in vestibular function [9].

For these reasons, it is well known that after VN, the ipsilateral VOR and its pathway are affected. This is evidenced, either by caloric, rotatory testing, vHIT, or VEMPs.

A vestibular lesion induces changes in the gain and time constant of the VOR system [10]. The gain of the VOR is an indication of the intensity of vestibular response and, therefore, of the vestibular compensation process. Some restoration and adaptation changes are usually described in these patients, a finding usually interpreted as a “compensatory system” [11].

Patients who suffer from vestibular neuritis tend to develop a persistent long-term disability in their daily activities. Some clinical variables such as the initial vestibular deficit, an enhanced visual dependence, or the level of anxiety may, to some level, predict outcomes in VN patients [12]. Although previous evidence reported that neither caloric test paresis nor VOR gain of vHIT predicts symptom outcomes in vestibular neuritis [13] other authors suggest that saccadic organization seems to participate in visual target retention and ocular compensation during head impulses in vHIT studies [14,15]. So far, there is no evidence of a relationship with vestibular function parameters, postural control, and perceived disability in vestibular neuritis.

The aim of this study is to analyze and compare the compensatory process, the evolution of vestibular dysfunction, postural control, and perceived disability in a population of patients with vestibular neuritis over time.

## 2. Materials and Methods

This was a longitudinal and prospective study. In all, 67 patients were recruited consecutively from the Department of Otorhinolaryngology in the University Hospital of Getafe from January 2017 to May 2020. Patients were admitted to the emergency unit due to acute vestibular syndrome and were finally diagnosed with vestibular neuritis. All patients were thoroughly assessed by a multidisciplinary team consisting of otorhinolaryngologists and a neurologist. Complete anamnesis, as well as otological and neurological examinations, were applied to every patient. In the emergency unit, all patients received the following treatment: prednisone (1 mg/kg in decrescent dose for 12 days) and antipsychotic (sulpiride 50 mg/8 h for maximum 3 days). After 3 months of follow up, when needed, vestibular rehabilitation therapy was offered to improve the imbalance.

The inclusion criteria for VN were (1) a single sudden onset of sustained vertigo, (2) unidirectional spontaneous horizontal nystagmus to the healthy side following Alexander’s law, (3) reduced caloric response (canal paresis > 25%) or VOR dysfunction in vHIT test, and (4) absence of neuro-otologic signs or symptoms suggestive of other peripheral, vestibular, or central disorders including hearing loss.

Exclusion criteria were (1) history of neurotologic disorders or otologic surgery, (2) medically ill condition or malignancy that can cause immunocompromised status, and (3) brain magnetic resonance imaging (MRI) that revealed acute infarction or other acute/chronic brain lesions, including cerebellopontine angle tumors.

This project was approved by the institutional review board (CEIm). Informed consent was obtained from every patient.

Patients received follow-up assessments 1 week, 1 month, 3 months, 6 months, and 1 year after the onset of vertigo. All tests necessary to analyze vestibular impairment and the process of recovery and compensation were performed from the initial visit until the end of the follow up. These included vHIT, cervical and ocular VEMPs, and bithermal caloric test. DHI and visual analogue scale were performed as well to know the disability perception of every patient.

Data were processed with SPSS version 22.0. For the evolution of continuous quantitative variables, the ANOVA test was used. Duncan’s post hoc test was used considering first-visit results as a reference. For qualitative variables, Chi^2^ test was performed. A study of multiple regression was performed to analyze the evolution of vHIT parameters, CDP, and disability questionnaires. Statistical significance for all tests was 0.05.

### 2.1. Vestibular Testing

Baseline vestibular responses were obtained with conventional bithermal caloric testing (30.5 °C and 43.5 °C) and a 10 s ice-water caloric test, when indicated. We used a video-based system (Ulmer VNG, v.1.4, SYNAPSIS^®^, Marseille, France) for the acquisition and analysis of the eye response. The maximum velocity of the slow phase of nystagmus evoked in each ear was analyzed to identify unilateral weakness and directional preponderance as determined by Jongkees’ formula. A mean unilateral paresis exceeding 25% indicated vestibular hypofunction.

We also evaluated the dynamic function of the horizontal semicircular canals using the vHIT (GN Otometrics; Copenhagen, Denmark). Fast, short, and unpredictable head impulses were performed in random horizontal directions while the subject was seated in front of the ground-fixed target and was instructed to maintain his/her vision continually fixed on the target during the test. Eye and head velocities were acquired with a sampling frequency of 250 Hz, and we calculated the hVOR gain from an average of 20 head impulses performed over a range of velocities from 100 to 250°/s.

To evaluate the function of vertical canals, the patient’s head was rotated 40° to the right to align it with the left-anterior–right-posterior plane. Patients were directed to continue staring at the same earth-fixed target as before. Brief, abrupt, forward and backward head impulses were made to stimulate the left anterior semicircular canal and the right posterior semicircular canal, respectively. After 20 impulses in each direction, the second pair of vertical canals was evaluated.

The presence or absence of saccades and their latency, and amplitude were registered for each exploration. To assess the dispersion of the saccades’ latency values, we used the PR score [16].

PR score is a quantitative variable that ranges between 0 and 100; when PR is close to 0, saccadic responses are described as gathered, and when it is nearing 100, they are described as scattered.

VEMP was performed for all patients using standard BERA equipment (SmartEP^®^ Intelligent Hearing Systems^®^). Each subject was tested while sitting down and turning their head away from the stimulated ear to contract the sternocleidomastoid muscle (SCM). The active electrode was placed in the middle of the upper third of the SCM and a reference electrode was placed on the chin. The ground electrode was placed on the forehead. Acoustic stimuli were presented through inserted earphones. Acoustic stimuli were 500 Hz tone bursts presented five times/second. The rise–plateau–fall time was 1–2–1. VEMP threshold and response amplitude were measured at a stimulus of 90 dB HL. The EMG from each side was amplified and bandpass filtered (10 Hz to 1.5 kHz). Results from 200 repetitions in each ear were averaged. The peak-to-peak amplitude (µV) was measured for P13–N23 potentials. To estimate the relative response in both ears, we used the interaural difference (IAD) ratio, calculated as (right ear amplitude − left ear amplitude) ÷ (right ear amplitude + left ear amplitude) × 100. A mean IAD ratio exceeding 40% indicated abnormal VEMP.

Computerized dynamic posturography (NeuroCom^®^ International, Inc., Clackamas, OR, USA) was carried out with the sensory organization test (SOT) battery. In this test, the patients were asked to maintain their balance under six different conditions. The first three conditions (SOT1, SOT2, and SOT3) provided accurate, uninterrupted foot support surface information. The visual information provided is different in each of these conditions. In SOT1, the patients keep their eyes open, whilst in SOT2, they must have their eyes closed. In SOT3, the patients must keep their eyes open, but the surroundings move in a pattern referred to by the patient antero-posterior (A-P) swaying movements. In SOT conditions 4, 5, and 6, the visual information is the same as those described for SOT1, 2, and 3 respectively, but the A-P sway movement of the patient drives the movement of the supporting surface in an axis parallel to the ankle joint. For every SOT condition, three trials were performed; in each of them, the A-P sway was measured and calculated relative to the sway of 12.5° (which is considered the maximum A-P sway about the ankle joint in normal subjects). In terms of general performance, a composite score (CS) was given as an overall estimate of postural stability, which is a weighted average of the results in different trials with special emphasis placed on the conditions SOT3 through SOT6.

### 2.2. Handicap Measurements

We used the DHI questionnaire [17], while vertigo severity was assessed using the visual analogue scale (VAS).

In the DHI, the patient had to answer “yes”, “sometimes”, or “no” to each question, the responses being designated 4, 2, and 0 points, respectively. The questionnaire had 25 items, so the total score ranged between 0 and 100.

VAS scores were obtained by asking the patient to rate the severity of their vertigo from 0 to 10.

## 3. Results

### 3.1. Demographic Data

A total of 67 patients participated in this study with a minimum follow up of one year. The mean age of patients was 52.53 ± 17.54 years. There were 33 (49.25%) females and 34 (50.75%) males, and 37 (55.22%) patients had a VN in the right ear.

The most prevalent comorbidities of patients were hypertension (39.7%), dyslipidemia (22.1%), type-2 diabetes mellitus (10.3%), and ischemic cardiopathy (5.9%).

The mean canal paresis was 55.37 ± 26.78 for the bithermal caloric test. Mean asymmetry was 70% ± 30% and 45.33% ± 30% for cervical and ocular VEMPs, respectively.

Mean values for horizontal, superior, and posterior canal of the vHIT were 0.45 ± 0.19, 0.29 ± 0.31, and 0.45 ± 0.17, respectively.

Based on the results of the vestibular tests, we could establish the affected branches of the vestibular nerve. In all, 42 (62.68%) and 4 (5.9%) patients had a single affection of the superior and inferior branch, respectively, while 5 (31.34%) patients had a total involvement of the vestibular nerve.

Mean values for canal paresis, cervical and ocular VEMPs asymmetry, and vHIT gain values depending on the affected branch are shown in Table 1.

Overall, 51 out of 67 patients (86%) had an abnormal CDP assessment. Looking at different subtypes, we identified 35 (52.85%) patients who showed a vestibular pattern where conditions 5 and 6 were primarily affected. A combination of a vestibular and visual preference pattern was observed in 10 (14.95%) patients. “Severity pattern” was observed in seven (10.44%) patients.

We observed a significant correlation between the composite score and baseline vestibular function elicited either by caloric, VEMPs, or vHIT test (*p* < 0.05).

Mean values for baseline VAS and DHI are shown in Table 2. There was a significant correlation between baseline vestibular function and first visit questionnaire scores (*p* < 0.05) and also between baseline composite score and first visit questionnaire scores (*p* < 0.05).

### 3.2. Gain and Saccades’ Evolution

Mean values for horizontal, superior, and posterior canal at each follow up are presented in Table 3.

When we compared every gain elicited in the different follow ups with the baseline, both horizontal and superior canal gains were significantly higher (*p* < 0.001) from one month until the last follow up.

**Figure 1:**
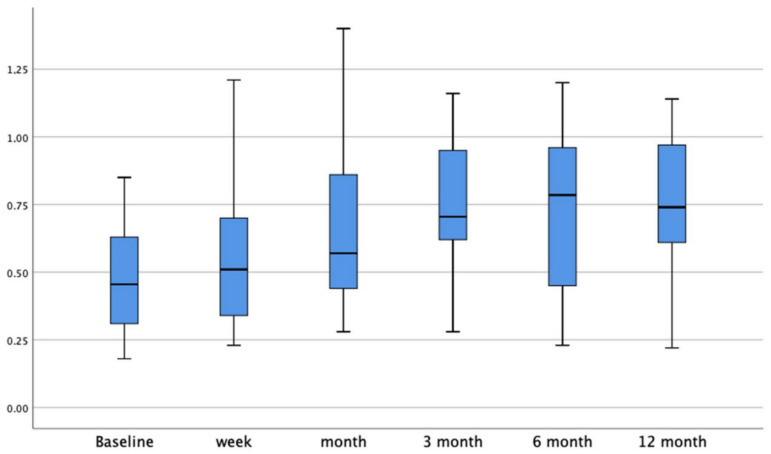
Bar graphic showing the HC gain values at every follow up.

The main gain recovery for the horizontal canal was 0.1 ± 0.04 for the first three months after the vestibular insult. After the 6-month follow up, the gain restoration significantly decreased to 0.01 ± 0.03 (*p* < 0.001). The HC gain bar graphic at every follow up is shown in Figure 1.

The main gain recovery for the superior canal was 0.1 ± 0.03 for the first three months after the vestibular insult. After the 6-month follow up, the gain restoration significantly decreased to 0.01 ± 0.049 (*p* < 0.01).

When we analyzed the evolution of the posterior canal gain in the global population, we did not observe a significant gain restoration (*p* > 0.05). If we excluded those patients with selective superior vestibular neuritis, we also observed a main gain recovery of 0.1 ± 0.029 for the first three months after the vestibular insult, with a significant decrease of such restoration after the 3-month follow up.

Table 4 shows the mean data for presence, latency, amplitude, and PR for both covert and overt saccades during the different follow-up periods.

The presence of covert and overt saccades significantly decreased after the 3-month and 6-month follow up, respectively, compared to the baseline results. The latency and amplitude significantly decreased after the 6-month follow up when compared to the baseline results.

**Figure 2:**
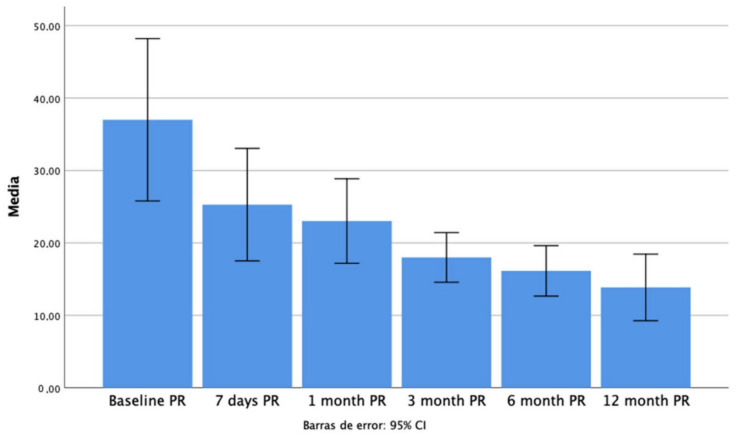
Bar chart for PR values at each follow up.

We observed a significant decrease in the overt saccades’ PR score from 3 months after the vestibular insult until the one-year follow up compared with initial data (Figure 2).

### 3.3. Postural Response Evolution

We observed a significant improvement in CDP’s conditions 5 and 6 and the composite score in the 6-month evolution compared to baseline (*p* < 0.001). We also observed a significant improvement (*p* = 0.01) in the anterior limits of stability in the same period.

We did not observe any further significant posturographic improvement from the 6-month follow up until the end of the study.

**Figure 3:**
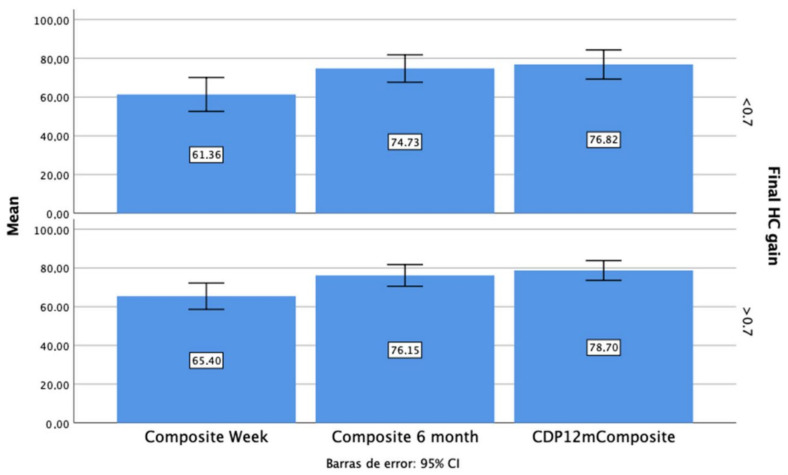
Composite score at every follow up, depending on the HC final gain.

As seen in Figure 3, those patients with a final HC gain below 0.7 had worse composite scores at every follow up, but those differences were not significant (*p* > 0.05).

### 3.4. Handicap Measurement Evolution

Regarding DHI and VAS measurement, we observed a significant decrease in both values from the first week until the last follow up, compared to the baseline measurement. When we compared each measurement with the previous one, we found a significant decrease in both values from the first-week visit until the 3-month follow-up measurement.

The variables DHI and VAS were found to be strongly positively correlated, r = 0.770, *p* < 0.001.

**Figure 4:**
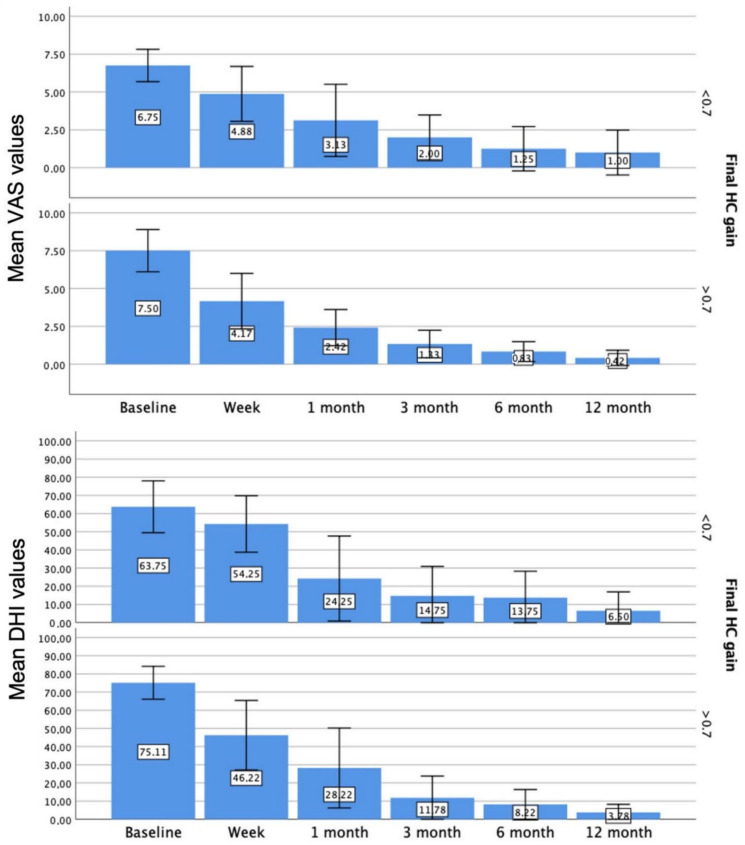
Bar chart for DHI and VAS score at every follow up, depending on the HC final gain.

As shown in Figure 4, those patients who finally obtained an HC gain above 0.7 had lower scores in either DHI or VAS measurement at every follow up (*p* < 0.05).

### 3.5. Relations of the Initial Damage with Vestibular Restoration, Postural Response, and Perceived Handicap

Those patients with an initial HC gain below 0.5 had significantly worse HC gain values and higher DHI and VAS scores at every follow up.

Additionally, those patients with an initial HC gain below 0.5 had a worse composite score at every follow up, but those differences were not significant (*p* > 0.05).

Regarding VEMPs measurement, the initial affectation of this myogenic potential predicted a worse HC gain at the 7-day follow up, without any further significant differences at ulterior follow ups. The initial caloric dysfunction did not significantly correlate either with the initial HC gain or the rest of the measurements elicited by vHIT during the different follow ups.

## 4. Discussion

This article shows how different easily obtainable measurements could become an objective and complete measurement of the state of compensation. This would give us information about the patient’s ability to abolish the residual symptoms after acute unilateral vestibulopathy and to recover postural control.

One of our findings was to show that ipsilateral-deficit VOR gains of the three semicircular canals constantly increased during the months following the onset of acute unilateral vestibular neuritis. Both the superior and the horizontal canals showed a decrease in the gain restoration after the 3-month follow up. In the case of vestibular neuritis, this gain improvement has two hypothesized components: central compensation and peripheral recovery.

The restoration resulting from the opposite vestibular nuclei is one of the key mechanisms of central compensation. This is achieved through biochemical events within the vestibular nuclei, and under specific conditions, strong synaptic plasticity may take place within the vestibular sensory organs. It is thought that this reactive plasticity can contribute to the repair of damaged contacts between hair cells and fibers of the vestibular nerve, thus gradually restoring peripheral sensory input [18].

As stated by other authors [19], the posterior canal’s function is affected significantly less by vestibular neuritis. However, in 4% of our patients, the PC was affected with an average gain of 0.45 ± 0.17, similar to the deficit in either the horizontal or superior canal. We can assume that changes in velocity VOR gains over time are equally produced in the three semicircular canals.

A decrease in the number of saccades and their latency and amplitude was also observed. We previously described [14] in a retrospective study, how the overtaking of the covert saccade is a sign of compensation. Many authors have reached similar conclusions [20,21] especially in populations where the VOR can change, indicating some marker of recovery of peripheral sensory function.

We also observed a significant decrease in this value regarding the PR score from 1 month after the vestibular insult until the 1-year follow up. We agree with other authors [22] that this measure has the potential to serve as a criterion in the follow-up evaluation of the vestibular compensation process after a vestibular insult.

We observed an improvement in the postural response, measured by a computerized dynamic posturography, either in the composite score or in the stability limits. As observed with the gain restoration, we did not observe any significant improvement after the 6-month follow up, a similar time window, whereby we should concentrate our efforts to improve postural control of our patients after a vestibular insult.

Along with either vestibular restoration or increase of postural control, our population also showed an improvement in the perceived disability, measured either by DHI or VAS, which were strongly correlated.

In our population, the final HC gain elicited one year after the vestibular insult may have a determinative clinical role since those patients with a final HC gain below 0.7 developed higher perceived disability and worse composite score at every follow up. Other authors [23] observed that six months of daily incremental vestibulo-ocular reflex adaptation training, resulted in a significant increase in the retained VOR gain during both passive and active head-impulse testing, along with a reduction of perception of disability.

In the same way, the main clinical predictor for those patients with worse vestibular compensation was the initial vestibular damage. Those patients with lower initial gain elicited by vHIT also developed significantly worse vestibular restoration and higher perceived disability, compared to those patients with less vestibular insult.

Thus, we consider the gain as a predictor of recovery; an important clinical clue that could lead us to manage our patients individually according to certain clinical parameters such as the initial damage or characteristics of the refixation saccades.

## 5. Conclusions

Different easily obtainable measurements could become an objective and complete measurement of the state of compensation. This would give us information about the patient’s postural control and disability at every follow up.

Our results provide us with a time window in which the vestibular restoration is still active and could give us clinical insights regarding the initial management of vestibular neuritis patients.

## Figures and Tables

**Table 1:** Mean values for canal paresis, cervical and ocular VEMPs asymmetry, and vHIT gain values, depending on the affected branch.

	Mean Canal Paresis
Bithermal caloric test	55.37 ± 26.78
	**Asymmetry**
cervical VEMPs	70% ± 30%
ocular VEMPs	45.33% ± 30%
	**VOR values**
vHIT horizontal canal	0.45 ± 0.19
vHIT superior canal	0.29 ± 0.31
vHIT posterior canal	0.45 ± 0.17

**Table 2:** Mean baseline values for baseline VAS and DHI.

	Mean ± SD
DHI (Functional)	27.14 ± 8.39
DHI (Emotional)	17.46 ± 7.73
DHI (Physical)	21.30 ± 7.38
DHI (Total)	66.70 ± 18.70
	**Mean ± SD**
Visual Analogue Scale	7.59 ± 1.85

**Table 3:** Mean values for the horizontal, superior, and posterior canal at each follow up.

	Initial	7 Days	1 Month	3 Months	6 Months	12 Months
Horizontal canal	0.45 ± 0.19	0.54 ± 0.25	0.69 ± 0.25	0.76 ± 0.23	0.79 ± 0.27	0.83 ± 0.23
Superior canal	0.29 ± 0.31	0.52 ± 0.21	0.62 ± 0.24	0.7 ± 0.2	0.74 ± 0.24	0.75 ± 0.22
Posterior canal	0.45 ± 0.17	0.69 ± 0.2	0.81 ± 0.21	0.78 ± 0.21	0.82 ± 0.19	0.8 ± 0.18

**Table 4:** Mean data for presence, latency, amplitude, and PR for both covert and overt saccades during the different follow-up periods.

COVERT Saccades	Initial	7 Days	1 Month	3 Months	6 Months	12 Months
Presence (%)	56.7%	68.8%	56.4%	50.0%	38.5%	40.8%
Latency (ms)	107.16 ± 30.32	107 ± 12.02	105 ± 19.57	104.11 ± 16.93	103.53 ± 9.64	100.2 ± 21.17
Amplitude (˚/s)	177.03 ± 56.31	170.5 ± 68.32	167.5 ± 53.31	137.78 ± 72.56	175.53 ± 80	150.45 ± 72.91
PR	22.69 ± 27.91	18.4 ± 13.26	22.86 ± 18.25	27.47 ± 17.74	29.72 ± 23.91	32.12 ± 28.47
**OVERT Saccades**	**Inicial**	**7 Días**	**1 Mes**	**3 Meses**	**6 Meses**	**12 Meses**
Presence (%)	92.5%	90.6%	84.6%	69.4%	74.4%	65.3%
Latency (ms)	218.02 ± 52.84	219.14 ± 62.13	203.3 ± 59.8	213.52 ± 53.02	211.3 ± 49.09	201.72 ± 53.25
Amplitude (˚/s)	217.8 ± 49.71	210.86 ± 57.21	178.09 ± 50.12	185.12 ± 50.47	187.69 ± 74.21	170.09 ± 66.39
PR	37.8 ± 12.2	25.7 ± 7.81	22.9 ± 6.93	18.1 ± 2.73	17.4 ± 2.91	13.4 ± 4.17

## Data Availability

Not applicable.
